# Foetal and neonatal alloimmune thrombocytopaenia

**DOI:** 10.1186/1750-1172-1-39

**Published:** 2006-10-10

**Authors:** Cecile Kaplan

**Affiliations:** 1 Unité d'Immunologie Plaquettaire, Institut National de Transfusion Sanguine, 6 rue Alexandre Cabanel, 75015 Paris, France

## Abstract

Foetal/neonatal alloimmune thrombocytopaenia (NAIT) results from maternal alloimmunisation against foetal platelet antigens inherited from the father and different from those present in the mother, and usually presents as a severe isolated thrombocytopaenia in otherwise healthy newborns. The incidence has been estimated at 1/800 to 1/1 000 live births. NAIT has been considered to be the platelet counterpart of Rh Haemolytic Disease of the Newborn (RHD). Unlike RHD, NAIT can occur during a first pregnancy. The spectrum of the disease may range from sub-clinical moderate thrombocytopaenia to life-threatening bleeding in the neonatal period. Mildly affected infants may be asymptomatic. In those with severe thrombocytopaenia, the most common presentations are petechiae, purpura or cephalohaematoma at birth, associated with major risk of intracranial haemorrhage (up to 20% of reported cases), which leads to death or neurological sequelae. Alloimmune thrombocytopaenia is more often unexpected and is usually diagnosed after birth. Once suspected, the diagnosis is confirmed by demonstration of maternal antiplatelet alloantibodies directed against a paternal antigen inherited by the foetus/neonate. Post-natal management involves transfusion of platelets devoid of this antigen, and should not be delayed by biological confirmation of the diagnosis (once the diagnosis is suspected), especially in case of severe thrombocytopaenia. Prompt diagnosis and treatment are essential to reduce the chances of death and disability due to haemorrhage. Due to the high rate of recurrence and increased severity of the foetal thrombocytopaenia in successive pregnancies, antenatal therapy should be offered. However, management of high-risk pregnancies is still a matter of discussion.

## Disease name/synonyms

Foetal/neonatal alloimmune thrombocytopenia (FAIT/NAIT) [1] or foeto-maternal alloimmunisation thrombocytopenia (FMAIT) [2].

## Definition/diagnostic criteria

Foetal/neonatal alloimmune thrombocytopaenia (NAIT) is a disorder caused by foetomaternal platelet incompatibility that usually presents as severe isolated thrombocytopaenia in otherwise healthy newborns. It results from destruction of the foetal platelets by maternal immunoglobulin G (IgG) antibodies elicited during pregnancy and directed against foetus-specific platelet antigens that are inherited from the father and are different from those present in the mother [1].

Clinically, the diagnosis is suspected when an otherwise healthy neonate, born after an uneventful pregnancy and delivery, exhibits petechiae or widespread purpura at birth or a few hours after birth. Visceral haemorrhages are less common. The mother is typically healthy, with no previous history of thrombocytopaenia, auto-immune disorders or ingestion of drugs. The infant has no clinical signs of infection or malformations (see Differential diagnosis). Approximately 20% of these infants show evidence of intracranial haemorrhage (ICH) leading to death or neurological sequelae (see Prognosis). The platelet count is low at birth and may be associated with anaemia, secondary to bleeding. Platelet immunological investigations will confirm the maternal specific alloimmunisation (see Diagnostic methods).

## Epidemiology

NAIT is the commonest cause of severe isolated thrombocytopaenia in the foetus and newborn. Prospective studies showed that it occurs in about 1 in 800 or 1000 live births [3,4]. Unselected cohort of neonates reported 0.9% frequency of neonatal thrombocytopaenia [5]. Immune aetiology was demonstrated in one third of these cases. As thrombocytopaenia when moderate (whatever its cause) is often silent, systematic neonatal blood sampling for a platelet count is the only possible way to detect neonatal thrombocytopaenia and to provide better management of the infant and subsequent pregnancies [5].

## Clinical description

In the foetus, alloimmune thrombocytopaenia is considered to be the most severe thrombocytopaenia. It may occur very early during pregnancy, and in several studies, ICH has been documented before 20 weeks of gestation [6-8]. In utero, ICH accounts for approximately 50% of the reported cases of ICH in NAIT. Therefore, NAIT should be considered as a potential aetiological factor in all cases where porencephaly, ventriculomegaly and, in fact, any type of ICH is discovered during the foetal life.

In the neonate, purpura or haematoma are the most common clinical manifestations. Visceral haemorrhages, such as gastrointestinal bleeding or haematuria, occur less frequently. ICH has been reported in NAIT (whatever the platelet antigen involved, see Aetiology) and is usually present at birth. ICH may also occur later, if the thrombocytopaenia persists. Mildly affected infants may be asymptomatic.

Anti HPA-1a and -3a immunisation induce severe neonatal thrombocytopaenia (see Aetiology) [9]. NAIT linked to HPA-5b incompatibility seems to be less severe than HPA-1a NAIT [10]. However, the infant may be symptomless, with thrombocytopaenia discovered incidentally, even in case of HPA-1a alloimmunisation. Therefore, unexpected or unexplained neonatal thrombocytopaenia or early onset of severe thrombocytopaenia in both pre-term and term babies should raise the possibility of NAIT and guide investigations accordingly.

## Aetiology

NAIT results from maternal immunisation against foetus-specific platelet antigens. The exact mechanism underlying maternal sensitisation remains unknown. A prospective study detected antibodies at 16 weeks of gestation in *primipara primigravida *women [3]. The maternal IgG alloantibodies can cross the placenta as early as the 14^th ^week of pregnancy. The foetal alloantigens are fully expressed as early as the 18^th ^week of gestation [11,12]. In these circumstances, foetal thrombocytopaenia (platelet counts below 150.10^9^/L) can occur very early during pregnancy and there is no spontaneous correction [3].

Although platelets express human leukocyte antigen (HLA) Class I and ABO blood group antigens at their surface, NAIT is mainly due to alloantibodies directed against platelet-specific alloantigens. A prospective study analysis showed that HLA antibodies did not cause thrombocytopaenia [14], unless there is an association with neutropaenia [15]. Casual observations suggest that NAIT is sometimes due to ABO incompatibility, although the particular features of these cases are not well established.

The so-called platelet-specific alloantigens, conventionally defined by their exclusive presence on the megakaryocyte lineage, have a phenotype frequency that varies between ethnic groups [16,17]. Since 1990, the Human Platelet Antigen (HPA) nomenclature has been adopted [18,19]. The platelet-specific antigen systems have been numbered chronologically in order of publication, and the allelic antigens designated alphabetically in order of frequency in the population: 'a' designating the higher frequency allele, 'b' the lower frequency allele. Polymorphism responsible for several of the platelet allotypes has been identified and a new nomenclature proposed [20]. Among the platelet-specific alloantigens, HPA-1a antigen is the form most commonly involved in NAIT in Caucasians [21], followed (at much lower frequency) by HPA-5b [10]. In Asians, NAIT is essentially linked to the HPA-4 system. Maternal immunisation against 'rare' or 'private' alloantigens has been reported [22-26].

Retrospective and prospective studies highlighted the importance of immunogenetic factors in platelet alloimmunisation. The HLA class II DRB3*0101 allele in mothers could be implicated in anti HPA-1a immunisation [27-29], whereas anti HPA-5b alloimmunisation was reported to be associated with a cluster of HLA DR molecules, sharing a particular polymorphic amino-acid sequence at position 69–70 in the DR**β**1 chain [30]. A better understanding of the immune response to platelet alloantigens would allow for a better definition and thus appropriate management of pregnant women at high risk.

## Diagnostic methods

The first step in the diagnosis is to confirm the isolated thrombocytopaenia in the newborn, and the absence of thrombocytopaenia in the mother. The platelet count is variable, usually as low at birth as <50. 10^9^/L. Anaemia occurs secondary to bleeding. Platelet immunological tests require expertise in the field. The therapy should be started as soon as a provisional diagnosis has been made. Any difficulties in confirming the diagnosis should not delay the therapy.

Testing involves the detection of maternal circulating antibodies and identification of the targeted platelet antigen, with determination of the platelet phenotype and genotype of both parents. Antibodies are usually detected with antigen capture enzyme-linked immunosorbent assay (ELISA) and the microplate enzyme-linked assay [31]. Molecular techniques are used for genotyping [32]. If the father is heterozygous for the considered antigen or if the paternity is uncertain, the platelet typing of the infant should be performed to confirm the diagnosis. Diagnosis is certain when parental antigen incompatibility with a corresponding maternal antibody is demonstrated. The biological diagnosis is unclear in the absence of such an antibody (in this case, a repeat testing may substantiate the diagnosis), or when a new, rare or private antigen is involved. Diagnosis should be unequivocally established before a subsequent pregnancy for a better management.

Diagnosis of foetal ICH is made by ultrasonography and magnetic resonance imaging (MRI).

## Differential diagnosis

Careful examination of the infant and consideration of the maternal history should exclude most of the other causes of neonatal thrombocytopaenia [33]. However, NAIT may also be associated with other causes of thrombocytopaenia, especially maternal antiplatelet autoimmunity [5]. 

### The main other causes of neonatal thrombocytopaenia :

#### Infection

Bacterial, viral or parasitic infections that may occur in intensive care units

#### Disseminated intravascular coagulation

Most often secondary to acute foetal distress or sepsis

#### Immune destruction

Maternal autoimmunity: autoimmune thrombocytopaenic purpura, lupus erythematosus; maternal use of drugs

#### Platelet consumption

Haemangioma, extensive thrombosis

#### Megakaryocytopoiesis impairment

• Chromosomal abnormalities

• Bone marrow metastases

• Congenital leukaemia

• Down-regulation of megakaryocytopoiesis during the course of Rh haemolytic disease or chronic hypoxia

#### Inherited causes

• Constitutional thrombocytopaenia such as Thrombocytopaenia associated with Absent Radii (TAR syndrome), Congenital Amegacaryocytopenia (CAMT), Wiskott-Aldrich Syndrome, Bernard Soulier Syndrome (BSS)

• Thrombocytopaenia due to inherited disorders such as von Willebrand 2B disease or constitutional deficiency in ADAMTS 13, leading to Thrombotic Thrombocytopaenic Purpura.

## Genetic counselling

The recurrence of thrombocytopaenia is very high and its severity usually increases in subsequent pregnancies. The risk depends on whether the father's platelet genotype is homozygous or heterozygous for the targeted antigen. Therefore, in case of heterozygosity or if the paternity is uncertain, the foetus platelet genotype must be determined with molecular biology techniques using amniocytes, microvilli or foetal blood sampling. Noninvasive typing from maternal blood is under investigation.

Platelet typing can be proposed to the sisters of an affected woman, in order to detect high-risk pregnant women. In our experience, antenatal management for the first affected pregnancy depends on the detection of maternal alloantibodies.

## Antenatal diagnosis

In cases of women already identified as at risk of having or developing HPA alloimmunisation (first child with NAIT or previous history of NAIT in the family), foetal genotyping may be performed either on chorionic villi or on amniotic cells.

Alloimmune thrombocytopaenia can be suspected in case of foetal ICH. Foetomaternal alloimmune thrombocytopaenia presenting antenatally as hydrops foetalis [34] has also been reported as a complication of foetomaternal platelet alloimmunisation. Recurrent miscarriages should be taken into account.

When incidental, thrombocytopaenia is discovered by foetal blood sampling and careful determination of contamination with amniotic fluid should be included. Considerations in the differential diagnosis of foetal alloimmune thrombocytopaenia include both maternal and foetal factors, among which thrombocytopaenia in foetuses small for date with the risk of intraventricular haemorrhage [35] and foetal hypoxia [36], infection, chromosomal abnormalities, but associations should not be ignored. In other causes of thrombocytopaenia, alloimmunisation should be studied when thrombocytopaenia is atypically severe.

## Management

### Neonatal management

Throughout the thrombocytopaenic period, the infant is at risk of haemorrhage, especially ICH. Optimal management should be initiated on the basis of the clinical situation, even before the diagnosis has been confirmed by platelet immunological testing.

#### Infants with haemorrhages or platelet counts below 30. 10^9^/L during the first 24 hours of life

The treatment of choice is the transfusion of platelets, which will not be destroyed by the maternal antibodies present in the circulation of the newborn. The mother is the best donor. After maternal transfusion, the infant's platelet count usually increases promptly, which itself argues in favour of alloimmunisation. The maternal platelets must be washed to remove the antiplatelet antibody and irradiated to prevent graft versus host disease. If the mother is not available, blood banks can provide HPA1b/1b donors' platelets, since HPA-1a incompatibility is the most frequent cause of NAIT. When private or rare platelet antigens are considered, members of the family can be genotyped. When compatible platelets are not available or their delivery is delayed, transfusion of random platelets with or without intravenous immunoglobulins may be performed [37]. Intravenous immunoglobulins alone should not be given in this situation, because of the delayed onset of their effect (18–24 hours after injection) [38].

#### Infants without haemorrhage and a platelet count above 30. 10^9^/L

These cases require close monitoring until an adequate platelet count is reached. Usually, platelet transfusion is not necessary, as the platelet count increases rapidly. Alternatively, in case of severe drop in the platelet count, the management described above should be considered. In some cases, intravenous IgG (1g/kg/day for 2 days) may be used to raise the platelet count.

#### Outcome and prognosis

The outcome depends on the severity of thrombocytopaenia at birth and the promptness of diagnosis and treatment. The need of immediate treatment depends on the presence of bleeding and the severity of thrombocytopaenia. If treatment is required, then it should not be delayed. In all cases, platelet counts should be closely monitored until a normal platelet count is obtained. The duration of the postnatal thrombocytopaenia is usually one to two weeks, but it may occasionally persist for longer. Radiological evaluation of the head (ultrasonography, MRI) is required to detect/exclude ICH. In absence of severe bleeding, the outcome is favourable. When ICH has occurred, retrospective studies in series of anti HPA-1a NAIT report a mortality of 10%, and a 20% rate of neurological sequelae [21,28,39].

### Management of subsequent pregnancies

The current management of pregnancies subsequent to a delivery of an affected child is aimed at preventing ICH during pregnancy and delivery. Currently, there is no techniques for evaluation the foetal status that do not involve invasive procedures (*i.e*. percutaneous umbilical blood sampling) and do not carry risks. However, in subsequent pregnancies, maternal anti-HPA-1a antibody titration has been recently shown to provide indications for the risk of severely affected foetuses (if measured in standardised conditions, before any therapy, and determined before 28 weeks of gestation) [40]. The optimal antenatal therapy to reverse foetal thrombocytopaenia is still a matter of debate [41-44]. As the results obtained by different teams in Europe [42] and in the USA [41] do not rely on randomised studies, conclusive recommendations cannot be provided. There is a consensus that women with high-risk pregnancies should be followed-up in referral centres (where they could receive antenatal therapy), with minimal use of invasive procedures. Maternal therapy including weekly maternal injection of high doses of immunoglobulins, with or without corticosteroids, is currently recommended as the first-line approach. Therapy can be stratified on the basis of the sibling history of NAIT [42-44]. Weekly intrauterine platelet transfusions with antigen negative platelets may be used as salvage therapy when maternal therapy has failed. Elective Cesaerean section is preferred when the foetus is thought to be severely affected.

## Unresolved questions

• Mechanism of maternal sensitisation

• Modulation of the maternal immunisation

• Real incidence of ICH

• Best antenatal management

• Setting up of the routine antenatal screening
